# Automated download and clean-up of family-specific databases for kmer-based virus identification

**DOI:** 10.1093/bioinformatics/btaa857

**Published:** 2020-10-08

**Authors:** Rosa L Allesøe, Camilla K Lemvigh, My V T Phan, Philip T L C Clausen, Alfred F Florensa, Marion P G Koopmans, Ole Lund, Matthew Cotten

**Affiliations:** National Food Institute, Technical University of Denmark, DK-2800 Kgs. Lyngby, Denmark; Novo Nordisk Foundation Center for Protein Research, Faculty of Health and Medical Sciences, University of Copenhagen, DK-2200 Copenhagen N, Denmark; National Food Institute, Technical University of Denmark, DK-2800 Kgs. Lyngby, Denmark; Department of Health Technology, Technical University of Denmark, 2800 Kgs. Lyngby, Denmark; Department of Viroscience, Erasmus University Medical Centre, 3000 CA Rotterdam, The Netherlands; National Food Institute, Technical University of Denmark, DK-2800 Kgs. Lyngby, Denmark; National Food Institute, Technical University of Denmark, DK-2800 Kgs. Lyngby, Denmark; Department of Viroscience, Erasmus University Medical Centre, 3000 CA Rotterdam, The Netherlands; National Food Institute, Technical University of Denmark, DK-2800 Kgs. Lyngby, Denmark; Department of Viroscience, Erasmus University Medical Centre, 3000 CA Rotterdam, The Netherlands; MRC/UVRI and LSHTM Uganda Research Unit, Entebbe, Uganda; MRC-University of Glasgow Centre for Virus Research, G61 1QH Scotland, UK

## Abstract

**Summary:**

Here, we present an automated pipeline for Download Of NCBI Entries (DONE) and continuous updating of a local sequence database based on user-specified queries. The database can be created with either protein or nucleotide sequences containing all entries or complete genomes only. The pipeline can automatically clean the database by removing entries with matches to a database of user-specified sequence contaminants. The default contamination entries include sequences from the UniVec database of plasmids, marker genes and sequencing adapters from NCBI, an *E.coli* genome, rRNA sequences, vectors and satellite sequences. Furthermore, duplicates are removed and the database is automatically screened for sequences from green fluorescent protein, luciferase and antibiotic resistance genes that might be present in some GenBank viral entries, and could lead to false positives in virus identification. For utilizing the database, we present a useful opportunity for dealing with possible human contamination. We show the applicability of DONE by downloading a virus database comprising 37 virus families. We observed an average increase of 16 776 new entries downloaded per month for the 37 families. In addition, we demonstrate the utility of a custom database compared to a standard reference database for classifying both simulated and real sequence data.

**Availabilityand implementation:**

The DONE pipeline for downloading and cleaning is deposited in a publicly available repository (https://bitbucket.org/genomicepidemiology/done/src/master/).

**Supplementary information:**

Supplementary data are available at *Bioinformatics* online.

## 1 Introduction

Next-generation sequencing is widely used and as a result, the amount of generated data has increased substantially. One common task is to classify sample data to identify possible origins. To do this, reference sequence databases are often used. Depending on the question addressed, a balance is sought between highly curated, reliable and yet limited reference sets versus more broadly inclusive reference sets that might contain misclassified or misassembled data. To establish a comprehensive database with as few problematic entries as possible is a critical step and has great impact on the results and conclusions made using these sets. In addition, for many agents such as RNA viruses, genomic changes over time can be extensive and using older reference genomes might not be optimal for novel strain identification. In addition, given the explosive growth of the use of next-generation sequencing, the publicly available datasets are growing fast. Thus, merely using an established reference database like the one provided by the National Center for Biotechnology Information (NCBI) might be suboptimal, compared to using all the available data from GenBank (NCBI Resource Coordinators, 2018), for discovery of new variants that have evolved over time (Goodacre *et al.*, 2018). Therefore, it would benefit the research community to be able to rapidly create new, clean and yet comprehensive databases from all publicly available data with easy updates to accommodate for the need for keeping up with increasing data availability.

NCBI’s GenBank has been an extremely valuable resource for sequence classification and the ever-growing, open access and inclusive nature of the database provide a wealth of information for the field. Because of the large number of entries, however, many redundancies exist. In addition, many entries contain contaminating sequences (e.g. host, vector) and improperly annotated entries (Holliday *et al.*, 2015). A simple download and use strategy, especially if taxonomy information is used, can lead to a high frequency of misclassifications. This is especially the case for read-based classification methods which, because of the limited information in a short read, can easily misclassify query sequences. Therefore, effort must be put into cleaning each of the downloaded sequences to facilitate classification and to avoid misclassifications in downstream analyses.

For viral discovery, the need for correctly documented and comprehensive reference databases is obvious and several exist for specific species or virus elements, such as HIV, influenza and repetitive elements (Bao *et al.*, 2015; Sharma *et al.*, 2015). However, the problems stated above particularly apply for virus discovery applications, as well as metagenomic diagnostics and surveillance (viromics), in which much broader databases are required and more comprehensive databases with most viral species and virus-associated species also exist (Brister *et al.*, 2015; Goodacre et al., 2018). New viruses are added to the public domain on a weekly basis, and state-of-the-art application of viromics, updated reference databases are essential to capitalize on this fast-growing new knowledge. Simple and automated methods to prepare either nucleotide or protein databases would be useful, and have the potential to provide a standard for laboratories seeking to move metagenomics into routine settings. The inclusion of protein sequences for viral identification may provide higher specificity and sensitivity to detect more distant relatives, which is essential for research applications (Cotten *et al.*, 2014; Ho and Tzanetakis, 2014).

We here present an automated workflow called Download Of NCBI Entries (DONE) with an easy update function for generating annotated and computationally well-structured databases by downloading all entries in either nucleotide or protein sequences from NCBI that match user-specified search strings. The workflow provides built-in options to add several cleaning steps including the removal of sequences matching appropriate predefined contamination entries. Furthermore, we show the applicability of this workflow on a case example with a virus database showing how the cleaning step can have a high impact on the classification results.

## 2 Approach

### 2.1 Automated download from NCBI

The DONE pipeline was created to be as user-friendly as possible by allowing database-specific changes and adjustments of both the download and structure of the database. At the same time, this approach is amenable to standardized searches for use in situations where accreditation is needed, like in diagnostic applications. This is achieved by a user-provided input file with each line containing the name of the database followed by a Boolean search string in the format used by NCBI’s GenBank. The number of raw entries downloaded will then correspond to the number of hits obtained when using the search string at the NCBI GenBank home page. To account for modifications in the database structure, DONE allows, in theory, an unlimited number of sub-databases specified as lines in the input file. The final database can then be a concatenated version of all sub-databases or kept as separate files. The final database will include a summary file keeping track of entries within each sub-database by saving information on which accession numbers belong to each database. This ensures ease of tracking of the content of each database and allows for subsetting the databases without having to download and create a new database.

The pipeline utilizes the UNIX-based Entrez Direct (Kans, 2019) to extract all accession numbers associated with each user-provided search string and then uses the URL-based E-utilities (Kans, 2019) for downloading GenBank files for each accession number. From each downloaded entry, information on available taxonomy and organism was extracted along with the actual sequence to provide additional information that can be included in the output when using the database. DONE supports the download of either nucleotide or protein sequences from the same input file, which is specified as a command line option. Within the download script, additional steps have been included to ensure all entries are downloaded even if internet connections are temporarily lost or unstable and to reduce the risk of overloading the NCBI server. These include keeping track of the number of entries compared to FASTA headers and downloading entries in batches.

To facilitate updates, the pipeline has an update function that compares newly extracted accession numbers from the same search string with accession numbers already included in the current version of the database. Consequently, only new entries will be downloaded when updating the database. In addition, entries that have been removed since the last download will be removed from the database. All changes will be written to a log file keeping track of the database history. The accession numbers from the old download will be kept to make it easy to switch back to an older version.

Including all genes and smaller subparts of a genome associated with a given taxonomy ID can often complicate downstream analysis. Therefore, it might be more optimal to limit the download to only complete or nearly complete genomes. To achieve this, we included an option for the user to add a ‘complete’ argument, that will add the keyword ‘complete’ to all search strings. However, given that the use of ‘complete’ in GenBank entries is not specific for ‘complete genome’; this does not guarantee to capture all complete genomes and many entries might be missed in the process. We tested other options for downloading only complete or nearly complete genomes, such as using length requirements and adding other keywords to the search string. The length requirements were based on viral genome/segment length information from Viral Zone (https://viralzone.expasy.org/). This was combined with a manual check of entry numbers as lengths were made more inclusive and a review of the literature was also made for select viruses. The accepted lengths included a buffer to include sequences 10% longer than the largest known genome (or segment) in the family and 10% shorter than the shortest genome (or segment) in the family.

Furthermore, additional search criteria may be beneficial to add, such as excluding sequences from patents (which are largely redundant) and certain species that often can be co-occurring, e.g. *Homo sapiens*. Standard arguments for the exclusion of patent and *H.sapiens* (by taxonomy ID) entries for all sub-databases have therefore been included as an argument in DONE. Other exclusion criteria can be added manually when generating the input file. The software code is available at https://bitbucket.org/genomicepidemiology/done/src/master/.

### 2.2 Cleaning of databases

**Fig. 1.**
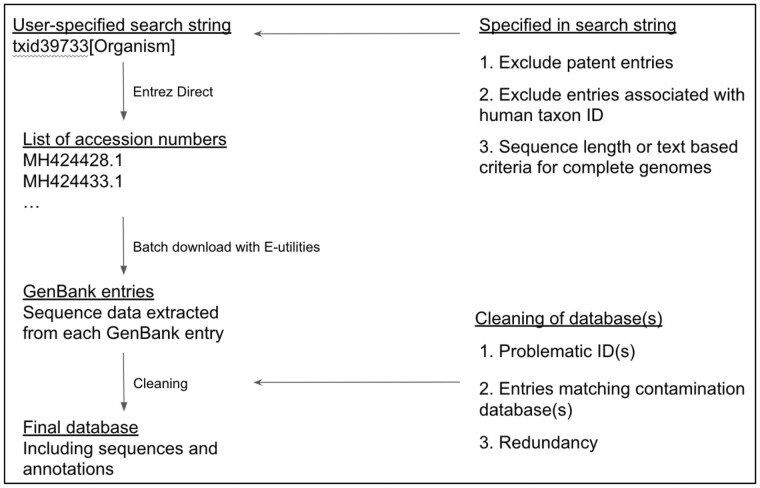
Overview of the automated download and cleaning process with DONE. Annotations include sub-database name, species and taxonomy ID

We tested the impacts of database cleaning. To do this, we included a series of cleaning steps as an extension of the DONE process. The first step of the cleaning was to exclude known problematic entries in an automatic manner. These include a set of GenBank entries that are known to be improperly annotated or include problematic sequence data. The accession numbers of these can be provided as a list in a separate file that can be expanded as knowledge grows. These lists can potentially be shared as they are being built by users. The second cleaning step was to remove any entries matching a sequence in a contamination database with a certain user-defined threshold. The contamination database can be any FASTA file with sequences that the user does not wish to include in the database. In the case of the virus database presented here, the contamination database consists of the UniVec database from NCBI (https://www.ncbi.nlm.nih.gov/tools/vecscreen/univec/), a bacterial plasmid database, a set of rRNA sequences and a collection of satellite sequences. A kmer-based nucleotide alignment algorithm, KMA (v1.2) (Clausen *et al.*, 2018), was applied to compute the alignment scores between each entry and the sequences in the contamination database. Consequently, the cleaning with the contamination database is currently only supported on nucleotides. The removal criteria were set to a percentage coverage of the contaminant. How to set this threshold and how it will impact the downstream analysis will be discussed in Section 3. The final step was to remove redundant entries from the database using USEARCH (v8.1) (Edgar, 2010). Furthermore, the user has the option to cluster sequences with a user-defined threshold if a further database reduction is desired. An overview of the download and cleaning process with DONE is shown in Figure 1.

### 2.3 Use of cleaned database for kmer-based viral identification

To demonstrate how the database with supplementary taxonomy and sub-database information can be applied, KMA was additionally used to build a kmer-based viral identification tool (KVIT). KVIT uses KMA with default mapping parameters but allows user’s inputs in parsing the output such as minimum identity and coverage criteria. The tool outputs text files with the best hits in the database and illustrates how the taxonomy can be used to show the composition of metagenomic samples on both species and family levels. KVIT has been implemented as an online web service that can be used with both assembled (FASTA format) and raw (FASTQ format) data at https://cge.cbs.dtu.dk/services/kvit/. Databases used for KVIT were all prepared and indexed with KMA using a kmer length of 16. In order to test the impact of cleaning, the DONE database and the accuracy of KVIT, a mock virus dataset was used containing short paired-end reads simulated from 18 different virus entries belonging to 5 different families, *E.coli* K12 along with one human chromosome (Supplementary Table S1). The dataset was constructed using wgsim (Li, 2019). Furthermore, KVIT was tested on real metagenomic samples with both the cleaned database downloaded with DONE and the NCBI viral reference database (RefSeq) (Brister *et al.*, 2015). The real metagenomic data were obtained from agnostically sequenced porcine fecal samples (Phan *et al.*, 2016).

## 3 Results

In this study, we developed a pipeline for automatic download and cleaning of reference sequences and showed the applicability of DONE by generating a virus database comprising 37 predefined virus families (see Supplementary Material BitBucket repository, https://bitbucket.org/genomicepidemiology/done/src/master/ for full list). To access the current development of each family in terms of amount of sequence data and contamination level, entries for each virus family were downloaded into separate sub-databases and concatenated into one final database post-cleaning. Initially, the search term for each sub-database included only the taxonomy ID(s) of that specific virus family. The Boolean search strings were then expanded by adding terms to exclude patents along with entries with an association to the human taxonomy ID (see Supplementary Material). The following results and discussion points will be based on variations of this database.

The full database download of all entries was completed June 7, 2019 and the size was 3.3 GB after concatenation of sub-databases. The largest contributor to download time and size was the Orthomyxoviridae database (which includes the influenza viruses) with a size of 1.1 GB, and the second-largest database was Herpesviridae at 525 MB. To assess the pace of data accrual, the databases were updated after approximately one month (July 8, 2019) and showed an increased database size with 16 776 new entries downloaded. Here, Orthomyxoviridae and Hepacivirus were the biggest contributors with 7542 and 3659 new entries, respectively. The average increase in entries per virus family was 453 per month. Therefore, the simple update implementation described here will be of great utility for maintaining fully up-to-date databases. If viral sequence data archives continue to grow at current pace, additional clustering of sequences to reduce the size may be necessary in the future to keep database size manageable.

Restriction to download to complete viral genomes for many research purposes, it is desirable to limit the database to only complete viral genomes rather than including all available genetic data, as this will both speed up the alignments and simplify downstream analyses. However, for some cases, it may be beneficial to include everything reported such as when dealing with rare species, or viruses subject to surveillance based on partial genomic data. Therefore, the opportunity to download everything is included as default in DONE to account for differences in usage and allow users to include their own specific filtering steps, if preferred.

When limiting the download to complete genomes only, a few options exist and are easily applicable using DONE. The preferred option is to set length criteria for each sub-database. However, this option requires prior knowledge of the species to be downloaded. Another option is to trust what is stated in the submission by only including hits with both ‘complete’ and ‘genome’ in the entry title. This can be further adjusted by allowing ‘partial’ as well, as it may include submissions with doubt about the actual completeness of the data even when assumed complete. The two options for downloading complete genomes mentioned above were tested on four virus families of different sizes; Coronaviridae (30 000 nt), Herpesviridae (250 000 nt), Picornaviridae (6–8 000 nt) and the Reoviridae (Segmented 200–3000 nt).

**Fig. 2.**
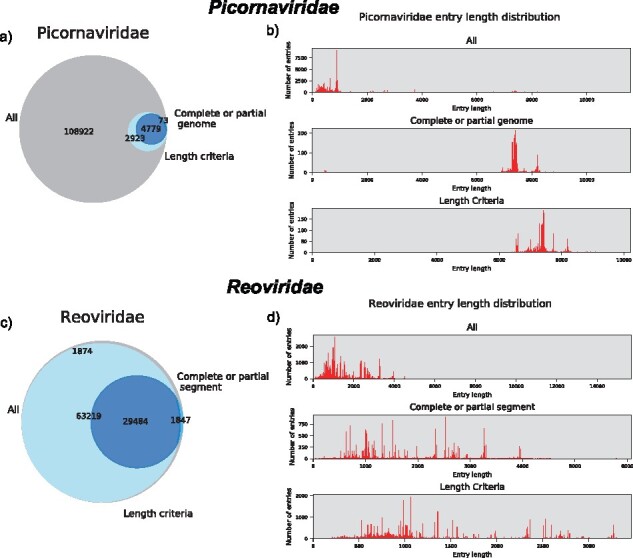
Results on three different downloading options for two virus families, Picornaviridae and Reoviridae, including all entries, entries with ‘complete’ or ‘partial’ in the description and entries within predefined length criteria specific for each virus family. (**a**) Venn diagram of entry overlap for Picornaviridae. (**b**) Length profile distributions for Picornaviridae. (**c**) Venn diagram of entry overlap for Reoviridae. (**d**) Length profile distributions for Reoviridae. The colors in (a) and (c) are as follows; Gray: ‘all’ +, ‘complete/partial’ -, ‘length’ -. Light blue: ‘all’ +, ‘complete/partial’ -, ‘length’ +. Blue: ‘all’ +, ‘complete/partial’ +, ‘length’ -. Dark blue: ‘all’ +, ‘complete/partial’ +, ‘length’ +

**Fig. 3.**
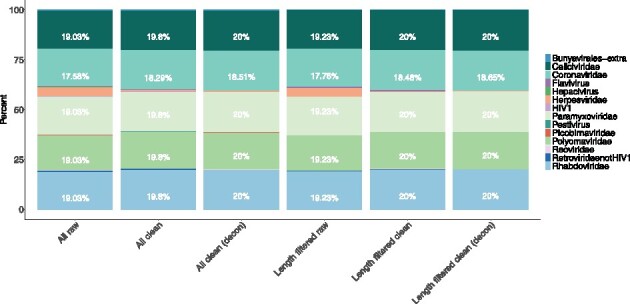
Percentage of mapped reads in the simulated viral metagenomic sample mapping to each of the sub-databases using the kmer-based alignment tool KVIT. The simulated sample contains equal amounts of reads for the five included viral families (Caliciviridae, Coronaviridae, Paramyxoviridae, Polyomaviridae and Rhabdoviridae) and additional contamination reads (phages, human and *E.coli*). Here, we show the distribution on a viral family level

Both the length and text-based criteria were introduced in the search string for each of the four sub-databases and downloaded with DONE. The download differences were then evaluated by comparing length profiles to the downloaded databases with and without the two criteria, as well as the actual entry overlap based on accession numbers. When downloading all entries, it is clear based on length profiles that the majority of downloads for the three non-segmented viral taxa were partial genome sequences (Fig. 2 and Supplementary Fig. S1). When limiting the download to full genomes another smaller peak around the size of the virus was identified. Especially for Picornaviridae, there was a large data reduction when applying the text-based (96%) or length criteria (93%). Here, the length criteria seem to include a few more entries than the complete/partial criteria, but they generally agree on which entries comprise full genomes (Fig. 1a and b). For the two larger vira, Coronaviridae and Herpesviridae, using the complete/partial and length criteria resulted in approximately the same reduction and the entries preserved are roughly the same (Supplementary Fig. S1). The main difference in the two approaches was found for Reoviridae where the length criteria included almost all data available (Fig. 3c and d). As Reoviridae have segmented genomes, the length criteria were more difficult to set due to large differences in lengths between segments so most data were included. From the complete/partial search term, almost 2/3 of the data was removed, indicating that not all entries from the length criteria are stated to contain a whole segment (Supplementary Table S2). Thus, the use of the length argument results in more comprehensive full genome or full segment databases. To create a more precise database of the segmented virus, a separate sub-database for each segment with the appropriate length requirements for that segment could be an easy solution. However, for this analysis, we decided to keep it simple and only include one sub-database for the segmented viruses. In conclusion, the length and complete/partial search term-based downloads showed similar results, except for the segmented Reoviridae. The complete database could be obtained in a more standardized manner across families by using the complete/partial search term instead of specific length criteria. However, the length criteria from our analysis tended to include more data than the complete/partial search term and it does not rely on the classification in the submission. Consequently, the database used for further analysis consisting solely of genomes or segments, referred to as the ‘length filtered database’ was downloaded based on the family-specific length criteria. For the ‘Unclassified’ virus family the ‘complete or partial genome’ search term was used, as an appropriate length criterion was not available.

When downloading the length filtered database, the total number of entries was reduced by 52% (from 1 880 052 to 905 203 entries) across all sub-databases, and the total size of the database was reduced by 32% (from 3.3 to 2.5 GB). The smaller reduction in size was primarily because small entries were removed, such as genes or targeted sequences. The decreased number of entries and size for each sub-database are shown in Supplementary Table S3.

### 3.1 Cleaning of the database

When downloading all available data without manual curation, some automatic cleaning steps will be highly beneficial for generating a trustworthy database. The downloads with DONE, both the ‘all’ database and the ‘length filtered’ database based on the length criteria, were cleaned using the described cleaning pipeline. Because entries were removed if they had too high similarity with a sequence in the database of known problematic sequences; it was necessary to determine a similarity threshold. Therefore, four different thresholds (0.5, 0.6, 0.75 and 0.95) for sequence identity with the contamination database were tested and the differences in the number of entries removed were evaluated for each virus family. The percentage of entries removed at each threshold is shown in Table 1 for the virus families with more than 0.5% of the entries removed at the lowest threshold for one of the databases. For the ‘all’ database only two families, Herpesviridae and Polyomaviridae, had more than 0.5% entries removed. When comparing the number of entries removed between the ‘all’ and ‘length filtered’ databases within each family there were not many extreme differences (Supplementary Table S4). This suggests that the entries with possible contamination sequences were generally observed in entries of partial or complete genomes. Consequently, only a small percentage of entries were removed from the ‘all’ database. Furthermore, it was clear that all removed entries mapped to the vector database.

**Table 1.** Percentage of entries removed at each similarity threshold against the contamination database of common contaminants

	All database				Length-filtered database			
Threshold	50%	60%	75%	95%	50%	60%	75%	95%
Herpesviridae	8.74	8.51	6.75	1.33	65.01	64.95	52.83	12.63
Papillomaviridae	0.16	0.0042	0.0042	0	0.61	0.017	0.017	0
Picornaviridae	0.051	0.042	0.033	0.00088	0.6	0.54	0.46	0.013
Polyomaviridae	1.1	1.1	1.1	0.41	2.18	2.18	2.18	1.27
Retroviridae (not HIV1)	0.2	0.16	0.097	0.026	3.35	2.91	1.26	0.62
Togaviridae	0.21	0.2	0.024	0.012	0.63	0.63	0.049	0

*Note*: We only list virus families for which more than 50% entries were removed at the lowest threshold (50%) in one of the databases. The ‘all’ database consists of every entry associated with the downloaded taxid. The ‘length filtered’ database includes length criteria on downloads specific for each database.

Based on these results, decreasing the threshold from 75 to 50% results in six sub-databases with more than 0.5% entries removed compared to the 3 with the 75% threshold. Therefore, it is important to consider the threshold when cleaning the databases and the best threshold might change depending on database usage. The Herpesviridae database had the most entries removed, and the number of entries removed did not change between the 50% and 60% thresholds. Furthermore, the amount of additional entries lost for the other families by choosing lower thresholds were limited between 50% and 60%, while the 75% and 95% thresholds had a bigger impact. Consequently, it was decided to use the clean database with a threshold of 50% for further analysis to ensure that the database did not contain any contamination even though it might result in decreased completeness of the database. We next set out to investigate the removed entries to check what they are and if they are indeed inappropriate. For Herpesviridae, the majority of the removed entries were from the same submissions and several were annotated as assemblies, which can contain errors or include non-related elements. Some removed entries were annotated as clones (e.g. MF468140) or specifically as unverified misassemblies (e.g. KX689266). In addition, for Picornaviridae, we observed some entries that contained replicons (e.g. AJ428955). Thus, we have manually confirmed that some of the entries are indeed appropriate to remove from the database prior to downstream analysis.

We decided not to include screening for human DNA contamination, as our approach completely removes hits above the coverage threshold. This could be a problem for viruses such as Retroviridae and Herpesviridae, as alignments to human sequences might actually be true positives. This complicates the analysis of real environmental samples, as human contamination could result in a false-positive detection and is one of the weaknesses of classification from short-read data. To deal with possible human contamination, we used a kmer-based masking approach implemented in KMA when using the database for species identification, which will be further assessed when using the database on simulated and real datasets.

The next part of the cleaning was to reduce redundancy, which was quite abundant (Supplementary Fig. S2). To further reduce the size of the database, clustering based on sequence similarities could be an option, however, we chose to construct a database with as much information included within reasonable criteria, as the level of diversity differs greatly among and within virus families and setting criteria would be a challenge. Especially, with the large variations within viral taxa keeping all but exact matches can be beneficial for further analysis and precise annotation. For our use of the database, we decided to only remove exact matches.

### 3.2 Results on simulated dataset

To further investigate how the downloaded databases can be utilized and what impact both the length requirements and cleaning have on usability, we tested the possibility to uniquely identify hits from a simulated sample of mixed viral genomes along with known usual contaminants, *E.coli* and human genomic DNA (Supplementary Table S1). The kmer-based identification tool, KVIT, was used together with each of the four final databases named ‘all raw’ (all entries without cleaning), ‘all clean’ (all entries with cleaning), ‘length filtered database raw’ (length-filtered database without cleaning) and ‘length filtered database clean’ (length-filtered databases with cleaning). The NCBI viral RefSeq database was downloaded on the same date as our final update (July 8, 2019) to compare the effects of using our databases with that of an established database. Generally, all four databases downloaded with DONE could be used for identifying the correct species and family and to identify the exact entry used to generate the simulated sample. However, some differences were observed between the databases regarding false-positive mappings and final identifications.

When using the two raw databases, 4.6% of the reads for the ‘all’ database and 4.2% for the ‘length filtered’ database mapped to Herpesviridae, which is not included in the simulated samples. Furthermore, some reads mapped to other databases such as Retroviridae (not HIV1), Hepacivirus and HIV1, but all with less than 1% for the ‘all raw’ database and less than 0.5% for the ‘length filtered raw’ database. When looking into the distributions of mapped reads, the ‘length filtered raw’ database seems to include less false-positive mappings than the ‘all raw’ database (Fig. 3). This indicates that the ‘all’ database contains small entries that might overlap with either the included families or the added contamination. When including the length criteria these are then removed.

For the ‘all raw’ database, the false-positive mappings lead to actual false-positive identifications above the coverage threshold for one sequence mapping to Herpesviridae and 20 mapping to Retroviridae (not HIV1), as seen in Table 2. For the ‘length filtered raw’ database none of the entries identified was above the coverage thresholds. When cleaning the ‘all’ database, the one Herpesvirus-designated entry was removed from the final result, but the 20 Retroviridae (not HIV1) hits remained and Herpesviridae still had a false-positive mapping of above 0.5%. This indicates that the contamination problem for the Retroviridae (not HIV1) is not removed in the cleaning step. A likely explanation is that this is caused by the presence of human genomic DNA.

**Table 2.** Additional hits identified with KVIT above the coverage threshold of 80% besides the known species included in the simulated sample

Family hit (entry name/species)	All raw	All clean	All clean (decon)	Length filtered raw	Length filtered clean	Length filtered clean (decon)
Herpesviridae (Stealth virus 1)	1	0	0	0	0	0
Retroviridae not HIV1 (Human Endogenous Retrovirus K, Multiple sclerosis associated retrovirus, Human endogenous retrovirus)	20	20	1	0	0	0

*Note*: The number states the unique entries identified for the family with the name of specific species listed for each family.

For the ‘length filtered clean’ database, the result was again slightly better than the ‘all clean’ database in terms of a higher percentage of mapped reads to the correct organisms (shown in Supplementary Fig. S3) and again only the correct hits were included in the result file.

To avoid false-positive assignments to human DNA, the KMA method used in KVIT was used to mask sequences based on a reference file and exclude them from the results. When using this decontamination option for the ‘all clean’ database against the human reference genome, 80 502 864 kmers mapped to the human genome corresponding to 57.44% of the database. For the ‘length filtered clean’ database 64 678 854 kmers mapped (58.10%). In a subsequent analysis with KVIT, the Retroviridae (not HIV1) hits were reduced to only one hit or not observed. The number of positive mappings to Herpesviridae was largely unchanged, suggesting that the hits were not a result of human contamination of the Herpesviridae. Given the repetitive sequence content of this virus family, an alternate possibility is that the positive hits are due to shared repetitive elements between material in the sample and the Herpesviridae references. For the ‘length filtered clean’ database, the decontamination option resulted in a small improvement with almost a perfect mapping distribution to the included organisms (Supplementary Fig. S3).

Consequently, these results highlight that more pre-processing is necessary for a database of all available entries without length requirements, while a length-filtered database in most cases can be used without additional cleaning if only hits with high coverage are accepted. We will recommend not using a database of all entries without the decontamination option or a similar approach to deal with human contamination in the input.

When comparing to the viral RefSeq database on species level, some false positives were identified compared to the databases downloaded with DONE (Supplementary Fig. S3). However, the correct identification of the known components was still achieved. One of the main differences is that the viral RefSeq database contains bacteriophages, which our database does not. The simulated dataset includes three types of phages, which explains why they are correctly identified in the sample using the viral RefSeq database and not using the DONE database. As expected both databases are highly useful for correct identification of components on a simulated dataset.

### 3.3 Results from a real case dataset

**Fig. 4.**
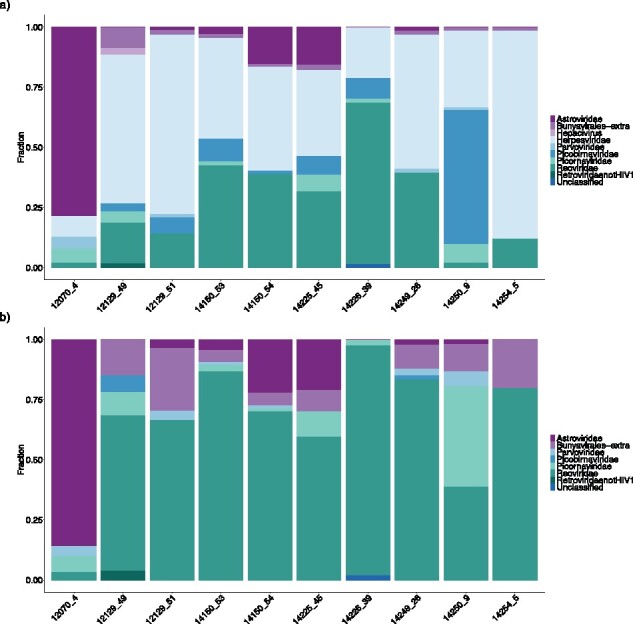
Distribution of the reads mapping to each sub-database on virus family level when using the kmer-based alignment tool KVIT on 10 real metagenomics samples from pigs. (**a**) Using the ‘all’ clean database with human decontamination. (**b**) Using the ‘length filtered’ clean database with human decontamination

In real applications, the contamination is most likely even more complex and an inappropriate cleaning of the database might lead to missing actual hits. Therefore, we tested how the two cleaned databases (‘all’ and ‘length filtered database’) differed in both distributions of mapping rates and final hits identified above an 80% coverage threshold on real metagenomic samples from pigs. We ran the analysis both with and without the decontamination option of both databases to evaluate whether possible mapping to Herpesviridae and Retroviridae could be human contamination. The results were compared to results obtained using the viral RefSeq database. This analysis has no ‘correct’ answer and, consequently, we can only compare differences observed when using the two approaches. For both ‘all clean’ (Supplementary Fig. S4a) and ‘all clean with decontamination’ (Fig. 4a), there were some issues presumably with wrongly identified Herpesviridae in most samples taking up almost all mapped reads except for sample 12070_4. From the results above, the coverage threshold the ‘all’ database did produce more hits (Supplementary Table S5), however, these come with some uncertainty due to the large amount of false-positive mappings to Herpesviridae. Nevertheless, even though many of the reads in the two versions of the ‘all’ database mapped to Herpesviridae, we only get one hit above the coverage threshold (Supplementary Table S5) without using the decontamination option. This supports that this database option can be useful with extensive cleaning and masking/removal of possible host sequences. For the length filtered databases, the contamination issue is absent indicating that small Herpesviridae entries are most likely the cause. The human decontamination again removed the Retroviridae mapping for the all ‘database’.

Based on these results, we would recommend using a ‘length filtered’ database with length criteria. This is especially the case if the database includes genomes of organisms with a high overlap with common contaminants and if a coverage threshold is not applied. If length criteria is not known, including ‘complete or partial’ in the search string can be an option, however, we do not recommend it. Again, using the NCBI viral RefSeq database indicating the presence of phages, and these were not included in the DONE database download. However, even without phages, the best hits were completely different looking at the exact entry level (Supplementary Fig. S5). Besides that, both the ‘length filtered’ and ‘all’ databases provided additional hits compared to the RefSeq database on an organism level (Supplementary Fig. S6). In addition, when looking at the hits with more than 80% coverage, the RefSeq database had little, if any, actual hits (Supplementary Table S5). This highlights that more precise and complete classifications can be obtained using a more comprehensive database.

## 4 Conclusion

Here, we show how a custom database can be prepared and maintained in an easy and user-friendly manner including fast update functions with detailed tracking information. Furthermore, we identify issues that can arise when using a partially uncurated database and how to deal with them in real applications for viral identification. The DONE pipeline for downloading, cleaning and updating a custom database requires only limited programming skills and combined with the KMA algorithm allows for additional cleaning with larger references. With some modifications to the download of length filtered data and establishing appropriate problematic sequence databases, DONE can be applied to create databases for other classification purposes such as for bacteria and eukaryotes. A similar analysis of the optimal cleaning procedure has to be completed to ensure the database is of good quality. Furthermore, the detailed tracking of included and removed entries makes the database easy to document and reproduce. Compared to other tools and databases, currently available DONE provides a more comprehensive cleaning and several easy options for customization of the database while still being reproducible and allowing fast comparison to other databases with a list of included accession numbers. This is highly useful in research where reproducibility and data sharing are becoming more troublesome with the high increase of new data.

## Supplementary Material

btaa857_Supplementary_Data
