# Supplementary material for: Automated download and clean-up of family-specific databases for kmer-based virus identification
Source: Bioinformatics. 2020 Oct 8;37(5):705–10. doi: 10.1093/bioinformatics/btaa857 (PMC8097684; doi:10.1093/bioinformatics/btaa857)
Supplement: btaa857_Supplementary_Data [file btaa857_supplementary_data.docx]

**Supplementary**


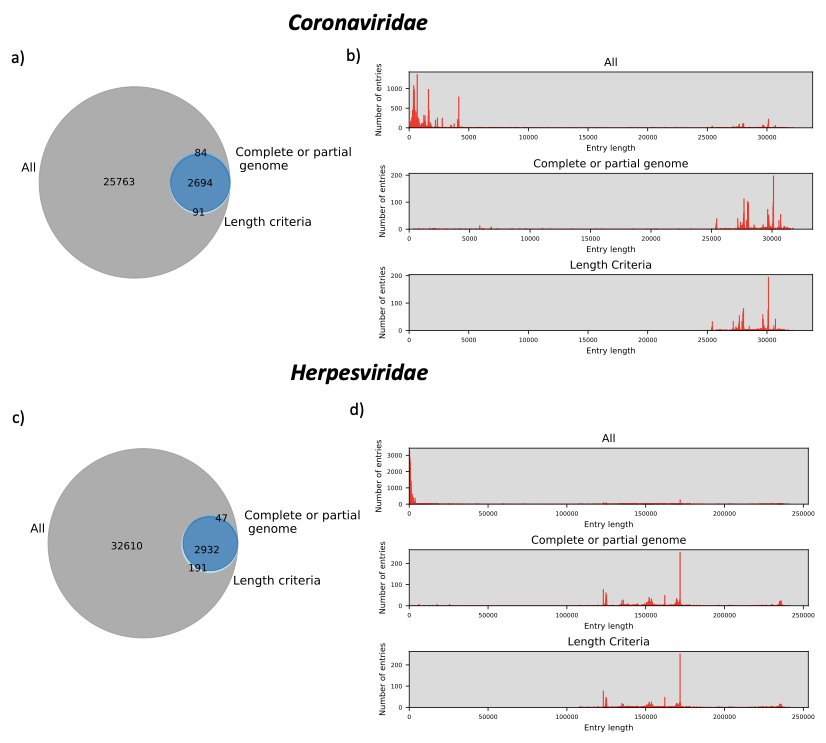


***Supplementary Figure 1:*** *Results on three different downloading options for two virus families, Coronaviridae and Herpesviridae, including 1. All entries (“All”), 2. Entries containing the terms “complete” or “partial” in the description (“Complete or partial genome”) and 3. Entries meeting a predefined genome length criteria specific for each virus family (“Length Criteria”). a) Venn diagram of entry overlap for Coronaviridae. b) Length profile distributions for Coronaviridae. c) Venn diagram of entry overlap for Herpesviridae. d) Length profile distributions for Herpesviridae. Please refer to Figure 1 for color annotation of the Venn diagrams.*


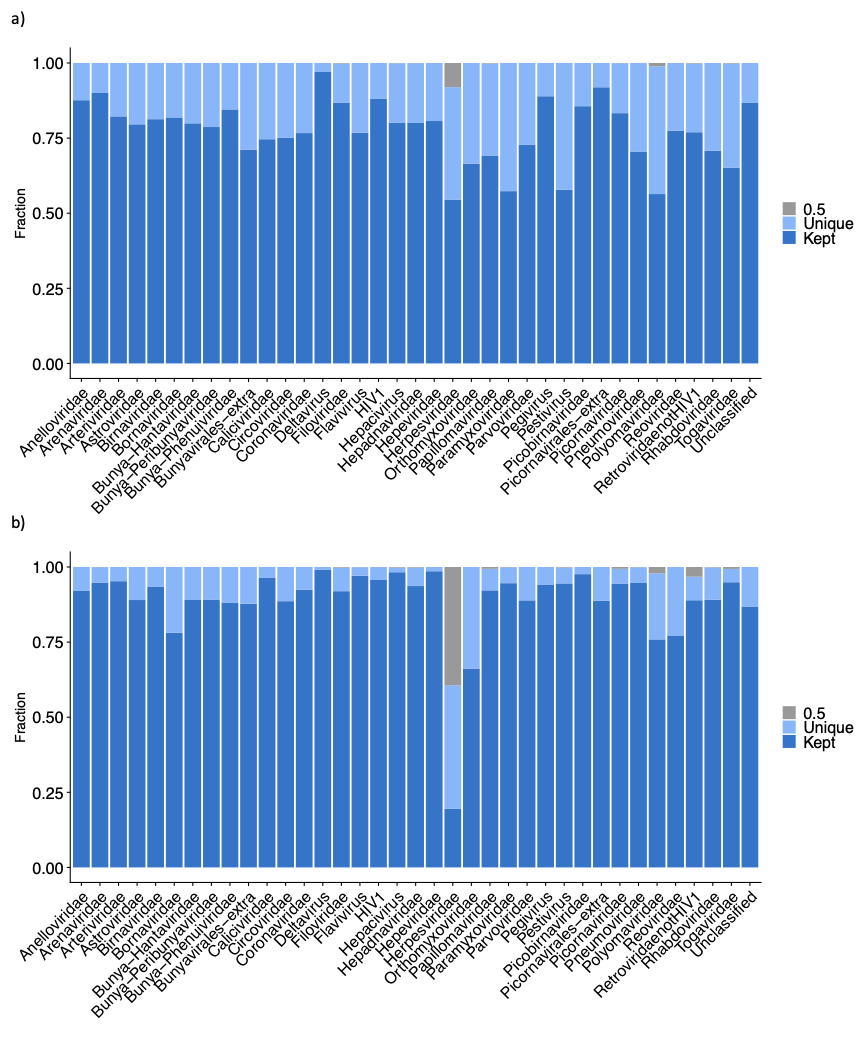


**Supplementary Figure 2:** *Fraction of entries removed by the final contamination similarity threshold of 0.5 and by unique criteria removing all redundant entries within a sub-database. a) Entries removed from the “all” database of every entry associated with the downloaded taxid. b) Entries removed from the “length filtered” database.*


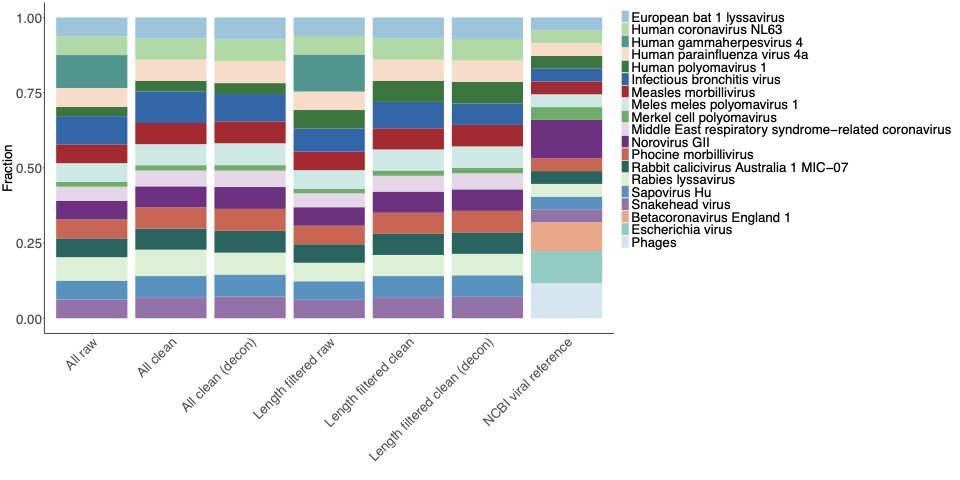
***Supplementary Figure 3:*** *Fraction of mapped reads in the simulated viral metagenomic sample mapping to each of the sub-databases using the kmer-based alignment tool KVIT. The simulated sample contains equal amounts of reads for the five included viral families (Caliciviridae, Coronaviridae, Paramyxoviridae, Polyomaviridae and Rhabdoviridae) and additional contamination reads (phages, human and E.coli) using wgsim. Here we show the distribution on a viral species level for each of the database constructions and the NCBI viral reference database. For the NCBI viral reference database, all identified bacteriophages were only reported as one group in the plot to reduce complexity.*


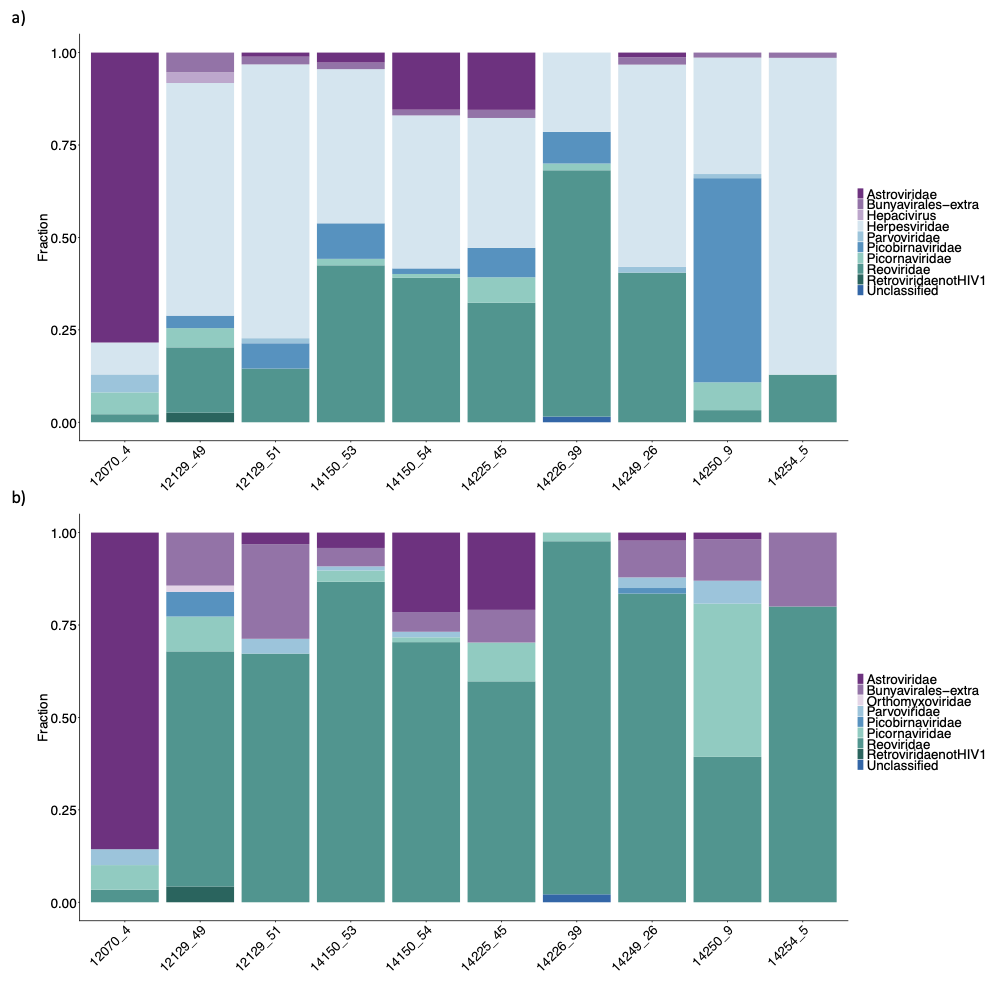


***Supplementary Figure 4:*** *Fraction of mapped reads in ten real metagenomic samples from pigs to each of the sub-databases using the kmer-based alignment tool KVIT. Here we show the distribution on a viral family level. a) Using the “all clean” database. b) Using the “length filtered clean” database*


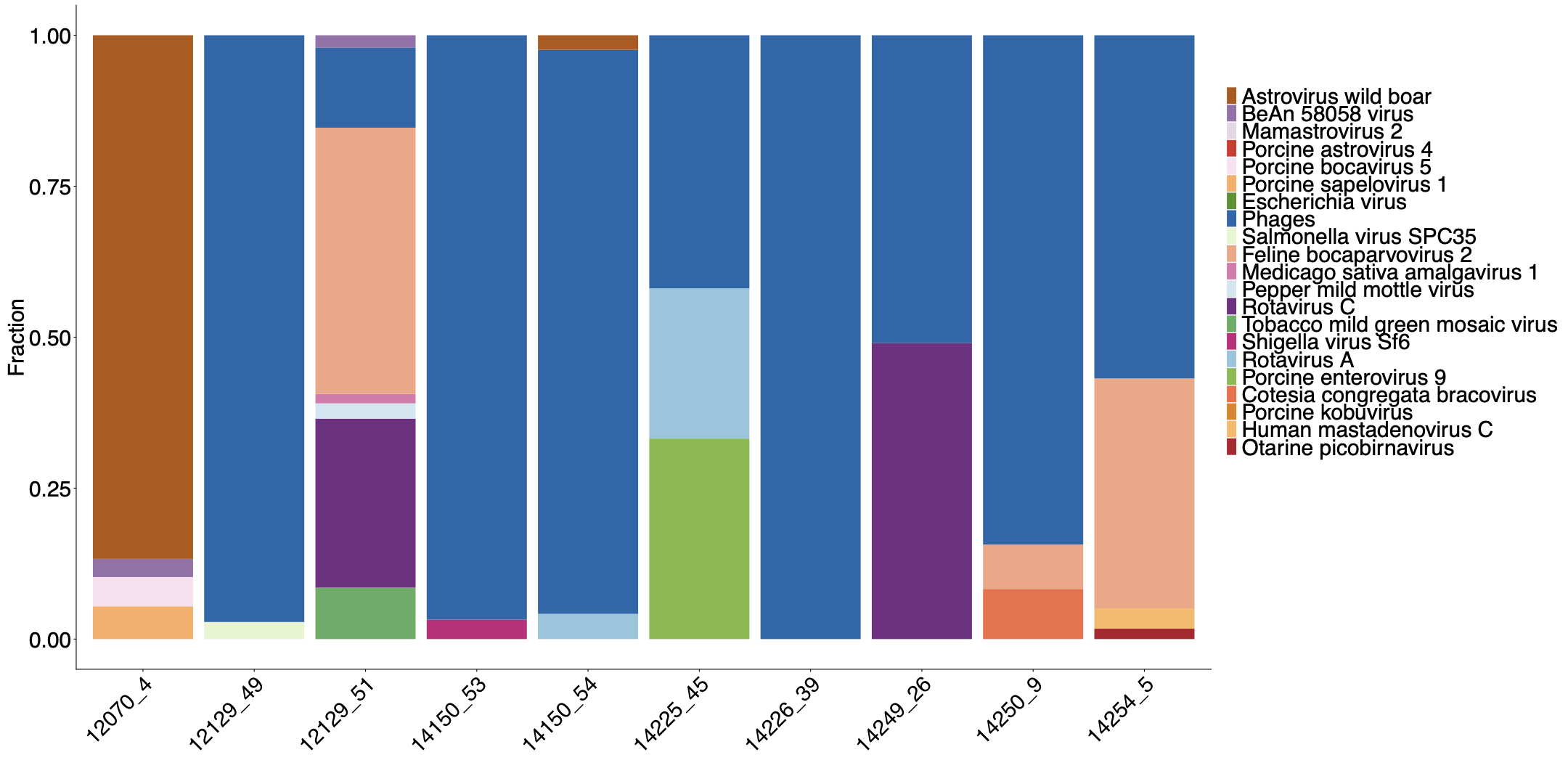


***Supplementary Figure 5:*** *Fraction of mapped reads in ten real metagenomic samples from pigs to the NCBI viral reference database using the kmer-based alignment tool KVIT. Here we show the distribution on a viral family level. Here phages were only included as one total count of all identified subtypes.*


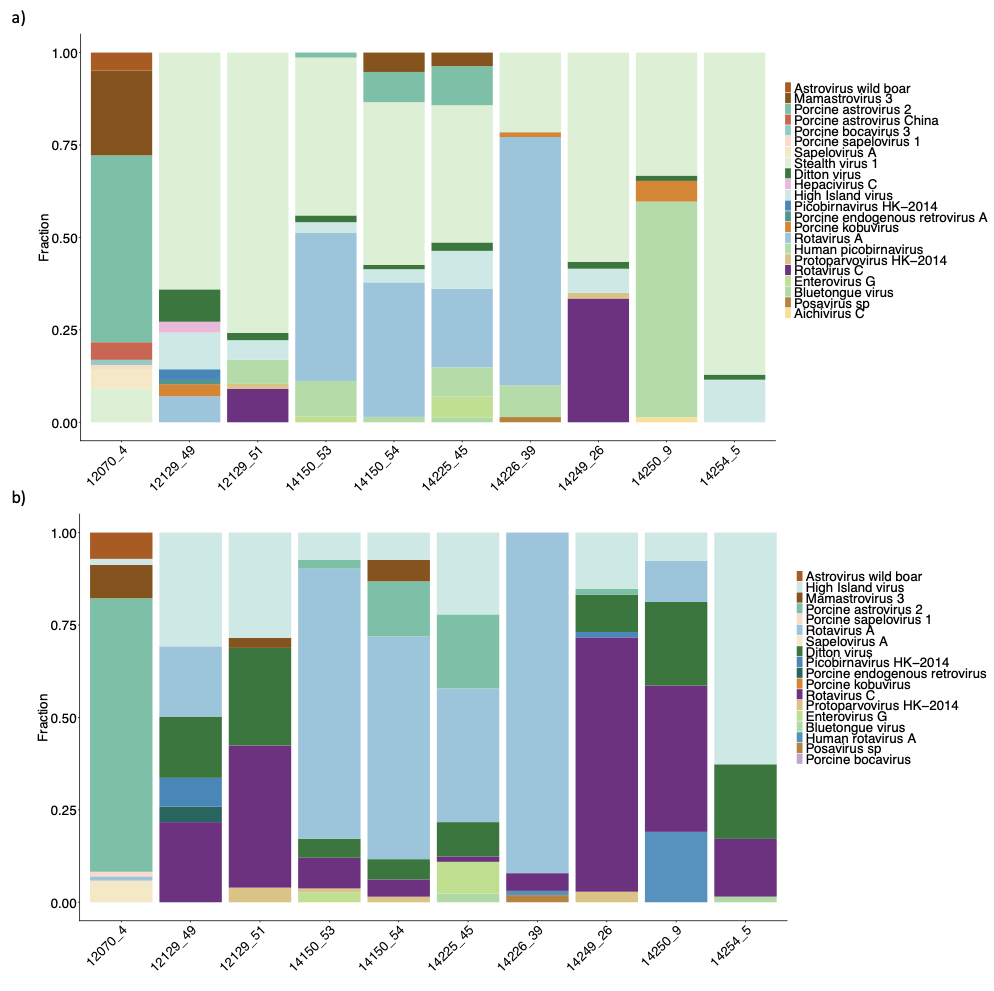


***Supplementary Figure 6:*** *Fraction of mapped reads in ten real metagenomic samples from pigs to each of the sub-databases using the kmer-based alignment tool KVIT. Here we show the distribution on a viral species level. a) Using the “all clean” database. b) Using the “length filtered clean” database*

| **Virus family** | **Entry 1** | **Entry 2** | **Entry 3** |
| --- | --- | --- | --- |
| ***Caliciviridae*** | KJ196293_GIIPe_GII4_Sydney2012 | Sapovirus_NC_006269 | Rabbit_calicivirus_NC_011704 |
| ***Coronaviridae*** | MERS_CoV_Al-Hasa_1_2013_KF186567 | Human_coronavirus_NL63_NC_005831 | Avian_infectious_bronchitis_virus_NC_001451 |
| ***Paramyxoviridae*** | Phocine_distemper_virus_NC_028249 | Measles_virus_NC_001498 | Human_parainfluenza_virus_4a_NC_021928 |
| ***Polyomaviridae*** | Merkel_cell_polyomavirus_NC_010277 | BK_polyomavirus_NC_001538 | Meles_meles_polyomavirus_1_KP644239 |
| ***Rhabdoviridae*** | Rabies_virus_NC_001542 | European_bat_lyssavirus_1_NC_009527 | Snakehead_rhabdovirus_NC_000903 |
| **Phages** | Acinetobacter_phage_AP205_NC_002700 | MS2_NC_001417 | Qbeta_NC_001890 |
| **Human** | H_sapiens_chr1_NC_000001 |  |  |
| ***E. coli*** | E_coli_K-12_NC_000913 |  |  |

**Supplementary Table 1:** *List of entries added to the simulated metagenomics sample. All entries contributed with 10,000 reads each except human and E.coli that contributed with 220,000 and 1,600,000 respectively.*

|  | All | Complete or partial | Length criteria |
| --- | --- | --- | --- |
| ***Coronaviridae*** | 28632 (107MB) | 2778 (75MB) | 2785 (77MB) |
| ***Herpesviridae*** | 35780 (525MB) | 2979 (460MB) | 3123 (489MB) |
| ***Picornaviridae*** | 116697 (126MB) | 4852 (35MB) | 7702 (55MB) |
| ***Reoviridae*** | 96424 (127MB) | 31331 (53MB) | 92703 (115MB) |

**Supplementary Table 2:** *Number of entries and database size for the four virus families used in the download test for complete genomes including all entries, complete or partial criteria in the description or family-specific length criteria.*

|  | **All database** | | **Length filtered database** | |
| --- | --- | --- | --- | --- |
| **Measurement** | **Size** | **Number of entries** | **Size** | **Number of entries** |
| ***Anelloviridae*** | 7.1MB | 8195 | 4.1MB | 1566 |
| ***Arenaviridae*** | 13MB | 3976 | 12MB | 2222 |
| ***Arteriviridae*** | 38MB | 29024 | 17MB | 1162 |
| ***Astroviridae*** | 7.1MB | 7997 | 2.6MB | 391 |
| ***Birnaviridae*** | 5.9MB | 5874 | 2.1MB | 723 |
| ***Bornaviridae*** | 1.4MB | 712 | 836KB | 93 |
| ***Bunya-Hantaviridae*** | 11MB | 8921 | 8.3MB | 3341 |
| ***Bunya-Peribunyaviridae*** | 11MB | 4893 | 9.7MB | 2454 |
| ***Bunya-Phenuiviridae*** | 13MB | 5753 | 11MB | 3088 |
| ***Bunyavirales-extra*** | 15MB | 10409 | 11MB | 2950 |
| ***Caliciviridae*** | 46MB | 46051 | 21MB | 2824 |
| ***Circoviridae*** | 13MB | 11173 | 9.6MB | 4858 |
| ***Coronaviridae*** | 107MB | 27924 | 78MB | 2795 |
| ***Deltavirus*** | 1.7MB | 2332 | 878KB | 525 |
| ***Filoviridae*** | 50MB | 3149 | 49MB | 2687 |
| **Flavivirus** | 144MB | 41704 | 144MB | 10731 |
| **HIV1** | 129MB | 218903 | 26MB | 3311 |
| **Hepacivirus** | 214MB | 100106 | 30MB | 10901 |
| ***Hepadnaviridae*** | 90MB | 14726 | 34MB | 634 |
| ***Hepeviridae*** | 11MB | 34471 | 4.4MB | 3125 |
| ***Herpesviridae*** | 525MB | 67715 | 490MB | 2957 |
| ***Orthomyxoviridae*** | 1.1GB | 720832 | 1.2GB | 714615 |
| ***Papillomaviridae*** | 57MB | 23771 | 45MB | 5949 |
| ***Paramyxoviridae*** | 70MB | 41741 | 41MB | 2768 |
| ***Parvoviridae*** | 23MB | 16598 | 10MB | 2159 |
| ***Pegivirus*** | 4.9MB | 5078 | 2.2MB | 234 |
| ***Pestivirus*** | 16MB | 21853 | 6.4MB | 547 |
| ***Picobirnaviridae*** | 2.1MB | 2128 | 1.3MB | 629 |
| ***Picornavirales-extra*** | 2.7MB | 396 | 2.5MB | 276 |
| ***Picornaviridae*** | 126MB | 113782 | 56MB | 7769 |
| ***Pneumoviridae*** | 54MB | 32374 | 33MB | 2251 |
| ***Polyomaviridae*** | 15MB | 10059 | 9.7MB | 1969 |
| ***Reoviridae*** | 128MB | 96642 | 116MB | 93562 |
| ***Retroviridae* (not HIV1)** | 138MB | 98245 | 14MB | 1580 |
| ***Rhabdoviridae*** | 53MB | 30791 | 26MB | 2236 |
| ***Togaviridae*** | 29MB | 8269 | 23MB | 1988 |
| **Unclassified** | 20MB | 3485 | 22MB | 3333 |

**Supplementary Table 3:** *Number of entries and size for each sub database when downloading all available hits and using the family specific length criteria.*

|  | **All database** | | | | **Length filtered database** | | | |
| --- | --- | --- | --- | --- | --- | --- | --- | --- |
| **Threshold** | **0.5** | **0.6** | **0.75** | **0.95** | **0.5** | **0.6** | **0.75** | **0.95** |
| ***Anelloviridae*** | 0 | 0 | 0 | 0 | 0 | 0 | 0 | 0 |
| ***Arenaviridae*** | 1 | 1 | 0 | 0 | 1 | 1 | 0 | 0 |
| ***Arteriviridae*** | 0 | 0 | 0 | 0 | 0 | 0 | 0 | 0 |
| ***Astroviridae*** | 0 | 0 | 0 | 0 | 0 | 0 | 0 | 0 |
| ***Birnaviridae*** | 0 | 0 | 0 | 0 | 0 | 0 | 0 | 0 |
| ***Bornaviridae*** | 0 | 0 | 0 | 0 | 0 | 0 | 0 | 0 |
| ***Bunya-Hantaviridae*** | 0 | 0 | 0 | 0 | 0 | 0 | 0 | 0 |
| ***Bunya-Peribunyaviridae*** | 0 | 0 | 0 | 0 | 0 | 0 | 0 | 0 |
| ***Bunya-Phenuiviridae*** | 0 | 0 | 0 | 0 | 0 | 0 | 0 | 0 |
| ***Bunyavirales-extra*** | 0 | 0 | 0 | 0 | 0 | 0 | 0 | 0 |
| ***Caliciviridae*** | 2 | 2 | 2 | 1 | 0 | 0 | 0 | 0 |
| ***Circoviridae*** | 0 | 0 | 0 | 0 | 0 | 0 | 0 | 0 |
| ***Coronaviridae*** | 0 | 0 | 0 | 0 | 0 | 0 | 0 | 0 |
| ***Deltavirus*** | 0 | 0 | 0 | 0 | 0 | 0 | 0 | 0 |
| ***Filoviridae*** | 6 | 6 | 6 | 0 | 6 | 6 | 6 | 0 |
| ***Flavivirus*** | 12 | 7 | 7 | 2 | 9 | 5 | 5 | 1 |
| ***HIV1*** | 7 | 6 | 6 | 0 | 1 | 1 | 1 | 0 |
| ***Hepacivirus*** | 159 | 153 | 154 | 105 | 7 | 2 | 2 | 1 |
| ***Hepadnaviridae*** | 45 | 44 | 39 | 12 | 20 | 20 | 20 | 3 |
| ***Hepeviridae*** | 0 | 0 | 0 | 0 | 0 | 0 | 0 | 0 |
| ***Herpesviridae*** | 3012 | 2932 | 2327 | 459 | 2033 | 2031 | 1652 | 395 |
| ***Orthomyxoviridae*** | 2 | 0 | 0 | 0 | 0 | 0 | 0 | 0 |
| ***Papillomaviridae*** | 39 | 1 | 1 | 0 | 36 | 1 | 1 | 0 |
| ***Paramyxoviridae*** | 2 | 2 | 2 | 0 | 2 | 2 | 2 | 0 |
| ***Parvoviridae*** | 0 | 0 | 0 | 0 | 0 | 0 | 0 | 0 |
| ***Pegivirus*** | 0 | 0 | 0 | 0 | 0 | 0 | 0 | 0 |
| ***Pestivirus*** | 1 | 1 | 1 | 0 | 1 | 1 | 1 | 0 |
| ***Picobirnaviridae*** | 0 | 0 | 0 | 0 | 0 | 0 | 0 | 0 |
| ***Picornavirales-extra*** | 0 | 0 | 0 | 0 | 0 | 0 | 0 | 0 |
| ***Picornaviridae*** | 58 | 48 | 38 | 1 | 47 | 42 | 36 | 1 |
| ***Pneumoviridae*** | 3 | 3 | 3 | 1 | 2 | 2 | 2 | 0 |
| ***Polyomaviridae*** | 111 | 111 | 103 | 41 | 43 | 43 | 43 | 25 |
| ***Reoviridae*** | 7 | 1 | 0 | 0 | 7 | 1 | 0 | 0 |
| ***Retroviridae (not HIV1)*** | 196 | 158 | 95 | 26 | 53 | 46 | 20 | 10 |
| ***Rhabdoviridae*** | 3 | 1 | 1 | 0 | 3 | 1 | 1 | 0 |
| ***Togaviridae*** | 17 | 17 | 2 | 1 | 13 | 13 | 1 | 0 |
| **Unclassified** | 0 | 0 | 0 | 0 | 0 | 0 | 0 | 0 |

**Supplementary Table 4:** *Number of entries* *removed for each cleaning threshold for similarity with sequence in the contamination database between for both the databases including all entries and database of complete genomes using a virus family specific length criteria.*

|  | All clean | All clean (decon) | Length filtered clean | Length filtered clean (decon) | NCBI viral reference |
| --- | --- | --- | --- | --- | --- |
| **12070_4** | | | | | |
| Mamastrovirus 3 | 7 | 6 | 1 | 1 | 0 |
| Porcine astrovirus China | 9 | 9 | 0 | 0 | 0 |
| Porcine astrovirus 5 (PoAstV 5) | 1 | 1 | 0 | 0 | 0 |
| Porcine astrovirus 4 | 14 | 15 | 0 | 0 | 0 |
| Porcine astrovirus 2 | 14 | 15 | 0 | 0 | 0 |
| Human alphaherpesvirus 1 (Herpes simplex virus 1) | 1 | 0 | 0 | 0 | 0 |
| Adeno-associated virus | 1 | 1 | 0 | 0 | 0 |
| Porcine bocavirus 3 | 3 | 3 | 1 | 1 | 1 |
| Bocavirus pig/7V/China/2010 | 1 | 1 | 0 | 0 | 0 |
| Porcine bocavirus | 3 | 3 | 1 | 1 | 1 |
| Human rotavirus A | 1 | 1 | 1 | 1 | 0 |
| Rotavirus A | 20 | 20 | 19 | 19 | 0 |
| Retroviridae | 5 | 0 | 0 | 0 | 0 |
| Human Endogenous Retrovirus K1,2-10 | 1 | 1 | 0 | 0 | 0 |
| Porcine bocavirus 5/JS677 | 0 | 0 | 0 | 0 | 1 |
| Rotavirus C | 20 | 20 | 19 | 19 | 0 |
| Rotavirus B | 20 | 20 | 19 | 19 | 0 |
| **12129_49** | | | | | |
| Rotavirus A | 11 | 11 | 11 | 11 | 0 |
| Aichivirus C | 1 | 1 | 0 | 0 | 0 |
| Enterobacteria phage RB49 (RB49) | 0 | 0 | 0 | 0 | 1 |
| Rotavirus C | 11 | 11 | 11 | 11 | 0 |
| Rotavirus B | 11 | 11 | 11 | 11 | 0 |
| **12129_51** | | | | | |
| Mamastrovirus 3 | 4 | 3 | 1 | 1 | 0 |
| Rotavirus A | 12 | 12 | 12 | 12 | 0 |
| Picobirnavirus PREDICT_PbV-101 | 1 | 1 | 0 | 0 | 0 |
| Rotavirus C | 12 | 12 | 12 | 12 | 0 |
| Bluetongue virus | 1 | 1 | 1 | 1 | 0 |
| Posavirus sp | 0 | 0 | 1 | 1 | 0 |
| Tobacco mild green mosaic virus | 0 | 0 | 0 | 0 | 1 |
| Rotavirus B | 12 | 12 | 12 | 12 | 0 |
| Picobirnavirus sp | 1 | 1 | 0 | 0 | 0 |
| **14150_53** | | | | | |
| Mamastrovirus 3 | 1 | 1 | 0 | 0 | 0 |
| Porcine astrovirus 4 | 3 | 3 | 0 | 0 | 0 |
| Porcine astrovirus 2 | 3 | 3 | 0 | 0 | 0 |
| Rotavirus A | 34 | 34 | 32 | 32 | 0 |
| Aichivirus C | 1 | 1 | 0 | 0 | 0 |
| Rotavirus C | 34 | 34 | 32 | 32 | 0 |
| Enterovirus G | 8 | 8 | 1 | 1 | 0 |
| Rotavirus B | 34 | 34 | 32 | 32 | 0 |
| **14150_54** | | | | | |
| Porcine astrovirus 4 | 1 | 1 | 0 | 0 | 0 |
| Porcine astrovirus 2 | 1 | 1 | 0 | 0 | 0 |
| Rotavirus A | 11 | 11 | 11 | 11 | 0 |
| Rotavirus C | 11 | 11 | 11 | 11 | 0 |
| Rotavirus B | 11 | 11 | 11 | 11 | 0 |
| **14225_45** | | | | | |
| Porcine astrovirus 4 | 1 | 1 | 0 | 0 | 0 |
| Porcine astrovirus 2 | 1 | 1 | 0 | 0 | 0 |
| Porcine bocavirus | 1 | 1 | 1 | 1 | 0 |
| Human rotavirus A | 2 | 2 | 2 | 2 | 0 |
| Rotavirus A | 18 | 18 | 18 | 18 | 1 |
| Aichivirus C | 1 | 1 | 0 | 0 | 0 |
| Rotavirus C | 18 | 18 | 18 | 18 | 1 |
| Bluetongue virus | 1 | 1 | 1 | 1 | 0 |
| Enterovirus G | 14 | 14 | 1 | 1 | 0 |
| Rotavirus B | 18 | 18 | 18 | 18 | 1 |
| uncultured picobirnavirus | 2 | 3 | 0 | 0 | 0 |
| Human picobirnavirus | 1 | 1 | 1 | 1 | 0 |
| Porcine enterovirus 9 | 3 | 3 | 0 | 0 | 0 |
| Porcine rotavirus A | 1 | 1 | 1 | 1 | 0 |
| Escherichia virus FI | 0 | 0 | 0 | 0 | 1 |
| **14226_39** | | | | | |
| Porcine bocavirus | 1 | 1 | 1 | 1 | 0 |
| Human rotavirus A | 3 | 3 | 2 | 2 | 0 |
| Rotavirus A | 19 | 19 | 18 | 18 | 0 |
| Aichivirus C | 1 | 1 | 0 | 0 | 0 |
| Rotavirus C | 19 | 19 | 18 | 18 | 0 |
| Bluetongue virus | 1 | 1 | 1 | 1 | 0 |
| Posavirus sp | 1 | 1 | 1 | 1 | 0 |
| Rotavirus B | 19 | 19 | 18 | 18 | 0 |
| Human picobirnavirus | 1 | 1 | 0 | 0 | 0 |
| Porcine rotavirus A | 1 | 1 | 1 | 1 | 0 |
| Sapovirus Hu/Kolkata/J20816 | 1 | 0 | 0 | 0 | 0 |
| Porcine kobuvirus/NLD45/2008/Netherlands | 1 | 1 | 0 | 0 | 0 |
| Picobirnavirus sp | 0 | 1 | 0 | 1 | 0 |
| Pacific flying fox faeces associated gemycircularvirus-13 | 0 | 0 | 1 | 1 | 0 |
| Porcine feces associated IAS virus like virus | 0 | 0 | 1 | 0 | 0 |
| Pteropus associated gemycircularvirus 4 | 0 | 0 | 0 | 0 | 1 |
| **14249_26** | | | | | |
| Rotavirus A | 14 | 14 | 14 | 14 | 0 |
| Rotavirus C | 14 | 14 | 14 | 14 | 0 |
| Bluetongue virus | 1 | 1 | 1 | 1 | 0 |
| Enterovirus G | 2 | 2 | 0 | 0 | 0 |
| Rotavirus B | 14 | 14 | 14 | 14 | 0 |
| uncultured picobirnavirus | 1 | 2 | 0 | 0 | 0 |
| **14250_9** | | | | | |
| Rotavirus A | 12 | 12 | 12 | 12 | 0 |
| Aichivirus C | 1 | 1 | 0 | 0 | 0 |
| Rotavirus C | 12 | 12 | 12 | 12 | 0 |
| Bluetongue virus | 1 | 1 | 1 | 1 | 0 |
| Rotavirus B | 12 | 12 | 12 | 12 | 0 |
| Porcine kobuvirus H15/2012/USA | 1 | 1 | 0 | 0 | 0 |
| Porcine sapovirus | 0 | 1 | 0 | 0 | 0 |
| **14254_5** | | | | | |
| Mamastrovirus 3 | 1 | 1 | 1 | 1 | 0 |
| Rotavirus A | 10 | 10 | 9 | 9 | 0 |
| Rotavirus C | 10 | 10 | 9 | 9 | 0 |
| Bluetongue virus | 1 | 0 | 1 | 1 | 0 |
| Rotavirus B | 10 | 10 | 9 | 9 | 0 |

**Supplementary Table 5:** *Result when running KVIT on the pig samples with a threshold of 80% coverage for accepting a mapping to the reference database. Here counting the number of accession numbers for each identified species in the sample across the different reference databases. The databases are the clean and decontamination version of all available data, the clean and decontamination version of the complete genome database and the NCBI viral reference database.*
